# Reliability, Validity, and Factor Structure of Pittsburgh Sleep Quality Index in Community-Based Centenarians

**DOI:** 10.3389/fpsyt.2020.573530

**Published:** 2020-08-31

**Authors:** Chi Zhang, Hao Zhang, Minghao Zhao, Zhongquan Li, Chad E. Cook, Daniel J. Buysse, Yali Zhao, Yao Yao

**Affiliations:** ^1^Department of Education, Beijing Hospital, National Center of Gerontology, Beijing, China; ^2^Institute of Geriatrics Medicine, Chinese Academy of Medical Sciences, Beijing, China; ^3^Department of Healthcare Policy and Research, Weill Cornell Medicine, New York, NY, United States; ^4^School of Basic Medicine, Peking University Health Science Center, Beijing, China; ^5^School of Social and Behavioral Sciences, Nanjing University, Nanjing, China; ^6^Department of Orthopaedics, Medical School of Duke University, Durham, NC, United States; ^7^Psychiatry and Clinical and Translational Science, University of Pittsburgh School of Medicine, Pittsburgh, PA, United States; ^8^Central Laboratory, Hainan Hospital of Chinese PLA General Hospital, Sanya, China; ^9^Center for Healthy Aging and Development Studies, National School of Development, Peking University, Beijing, China; ^10^Center for the Study of Aging and Human Development and Geriatrics Division, Medical School of Duke University, Durham, NC, United States

**Keywords:** Pittsburgh Sleep Quality Index, reliability, validity, factor analysis, centenarians

## Abstract

**Background:**

The Pittsburgh Sleep Quality Index (PSQI) is a widely used self-report questionnaire that measures general sleep quality in general populations. However, its psychometric properties have yet to be thoroughly examined in longevous persons.

**Objectives:**

This study aimed to explore the reliability, validity and factor structure of the Chinese-language version of the PSQI in community-dwelling centenarians.

**Methods:**

A total of 958 centenarians (mean age = 102.8 years; 81.8% females) recruited from 18 regions in Hainan, China, completed the PSQI scale. Cronbach’s alpha coefficient was used to measure the internal consistency. Exploratory factor analysis (EFA) and confirmatory factor analysis (CFA) were performed to explore the validity and factor structure of the PSQI in this sample. Correlations between the global PSQI score and physical function, depression symptoms, self-reported health status and subjective well-being were used to assess divergent validity.

**Results:**

The Cronbach’s α coefficient of the PSQI was 0.68, and it increased to 0.78 after two components (medication use and daytime dysfunction) were removed. The Spearman correlation coefficients of the PSQI score with each component were statistically significant (*P*<0.01). EFA yielded a two-factor structure model of the original PSQI-7 and a one-factor structure model of the simplified PSQI-5. The one-factor model with five components (χ^2^/*df* =1.59, CFI=0.99, RMSEA=0.03) fit the data well and had good configural invariance across demographic characteristics (0.53<_Δ_χ^2^<5.58, *P*>0.05).

**Conclusions:**

The original PSQI showed acceptable applicability in Chinese community-dwelling centenarians, and its psychometric characteristics moderately improved after sleeping medication and daytime dysfunction were removed. Further validation studies on PSQI are needed among centenarians from varied backgrounds.

## Introduction

Sleep problems, including insufficient sleep, poor quality sleep, and sleep disorders such as insomnia and sleep apnea, are highly prevalent among older populations (1). Poor sleep quality increases the risk of falls (2, 3), obesity (4), cognitive dysfunction (5), cardiovascular diseases (6, 7), as well as neurologic or psychiatric conditions (8, 9). Thus, sleep quality has become an important index for evaluating the health and life status of older adults, especially for the vulnerable oldest-old populations (10). As oldest-old adults, including centenarians, constitute the fastest growing segments of the world population (11), their sleep quality warrants more attention. Although some studies indicated that centenarians represent a prototype of successful aging (12), poor sleep quality is relatively high in this population (13, 14). In addition, both the symptoms and influencing factors of sleep problems in long-lived populations have varied patterns compared to the symptoms and factors among younger generations (15, 16). Thus, it is imperative to verify tools for measuring sleep quality among this exceptionally aged population.

The Pittsburgh Sleep Quality Index (PSQI) (17), a widely used self-reported questionnaire, is considered to be a generic instrument to measure sleep quality in diverse populations (18, 19) and has been employed in several centenarian studies (20, 21). The PSQI covers a broad range of indicators relevant to sleep quality and its components (namely, subjective sleep quality, sleep latency, sleep duration, habitual sleep efficiency, sleep disturbances, use of sleeping medication, and daytime dysfunction) provide references for clinical decisions. A systematic review including 37 studies confirmed that the PSQI had a fair or good Cronbach’s alpha coefficient (ranging from 0.64 to 0.83) (17, 22). Previous studies about community-dwelling older adults demonstrated single-factor (23), two-factor (24) and three-factor models (25) with different methodologies. Published findings also showed adequate internal consistency (19) and construct validity but varying factor structures among the English, Chinese, Korean, Portuguese, Nigerian and Greek versions of the PSQI (26–30). A recent systematic review including 45 articles and demonstrated the heterogeneous dimensionality of PSQI in both clinical and community-dwelling populations (31). A cross-sectional study including 17 comparative factor structures explored the fitness of different PSQI factor scoring models (32). Moreover, some studies also reported conflicting results in older adults (33–35).

Although previous studies have explored various properties of the PSQI across different clinical and nonclinical groups (19), its applicability in long-lived populations is not clear. The objective of this study was to examine the reliability, validity and factor structure of the PSQI in a group of community-dwelling Chinese centenarians.

## Methods

### Participants

The sample of this study was obtained from the baseline survey of the China Hainan Centenarian Cohort Study (CHCCS), which was conducted in Hainan, China, from June 2014 to December 2016. Hainan province has one of the highest life expectancies and percentage of centenarians in China and was authorized by the International Expert Committee on Population Aging and Longevity as a “World Longevity Island” (36). The CHCCS was designed as a whole sample study; it aimed to evaluate centenarians’ physical and mental health status and identify modifiable factors related to aging and longevity.

**Figure 1:**
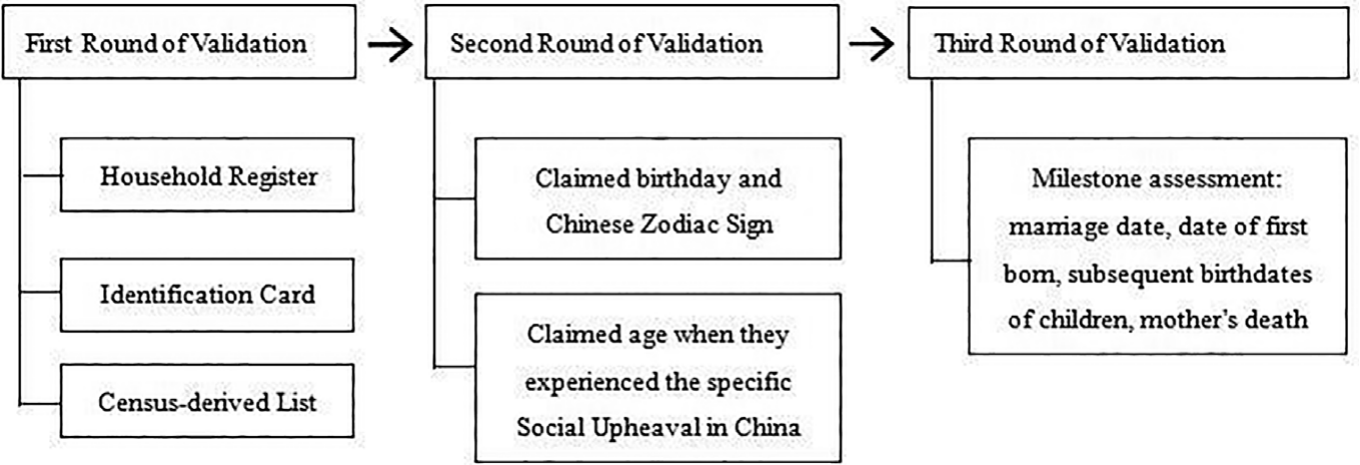
Three-steps age validation process of centenarians in China Hainan Centenarian Cohort Study (CHCCS).

**Figure 2:**
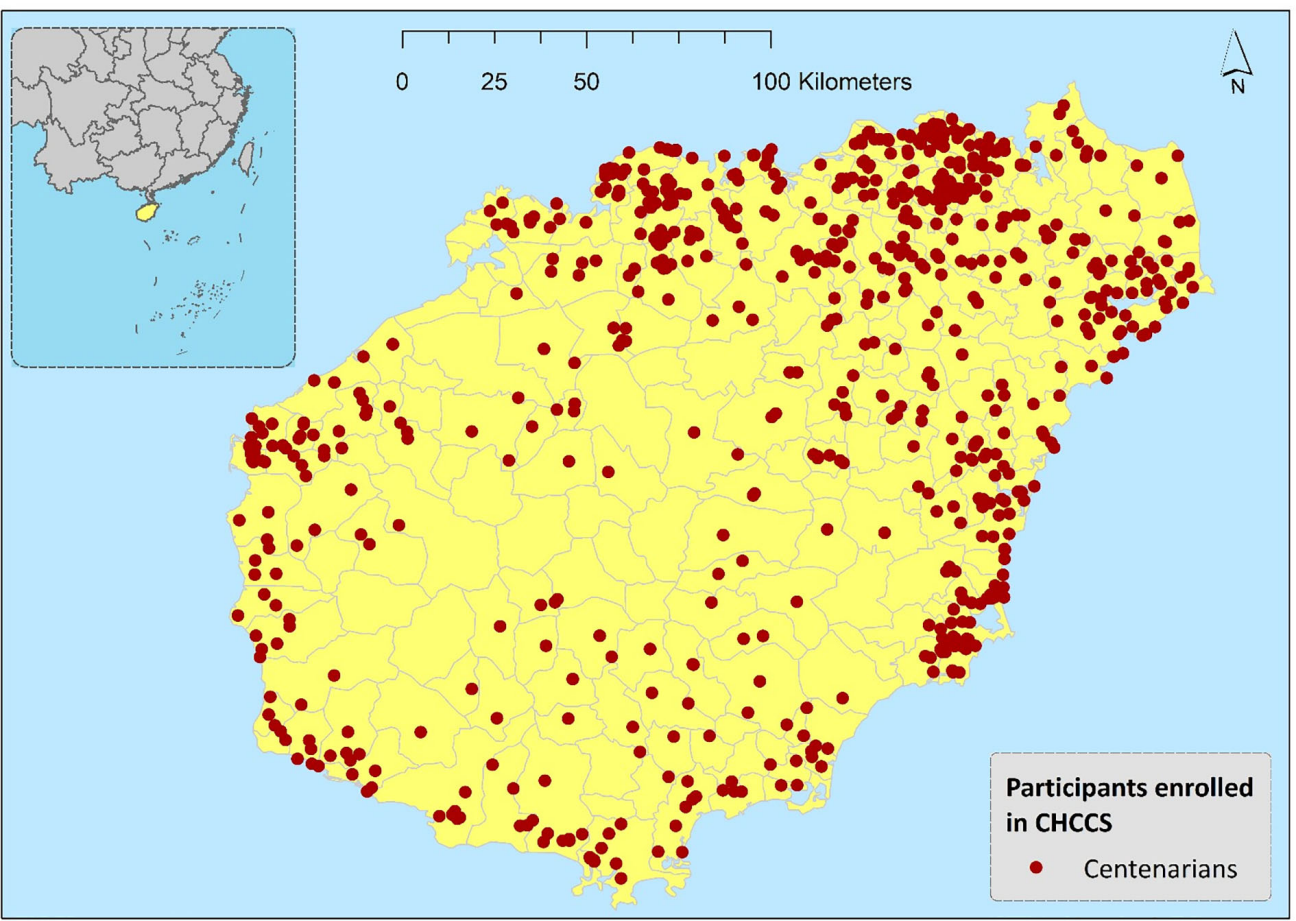
Geographical distribution of participants in China Hainan Centenarian Cohort Study (CHCCS).

Prior to the investigation, three steps of the age verification method were adopted to ensure the age authenticity of the enrolled participants; 58 participants failed the age validation (**Figure 1**). A total of 1002 centenarians were eventually included and surveyed in the CHCCS. Details of the sampling strategy and inclusion and exclusion criteria have been reported elsewhere (37). After further excluding 44 participants who failed to complete the questionnaires due to critical diseases, 958 participants (Mean age = 102.8 ± 2.7 years; 81.8% females) from all 18 regions throughout Hainan Province were included. The geographical distribution of participants is shown in **Figure 2** with red dots. The participants’ primary caregivers assisted in describing the details of the investigation. The ethics committee of the Hainan branch of the Chinese People’s Liberation Army General Hospital (Sanya, Hainan) approved the study protocol (no. of serial: 301hn11201601).

### Measures

#### The PSQI

The PSQI is an 18-item constructed questionnaire designed to assess overall sleep quality over a 1-month period. The 18 items are divided into 7 derived component scores: (1) sleep quality; (2) sleep latency; (3) sleep duration; (4) sleep efficiency; (5) sleep disturbance; (6) medication use; and (7) daytime dysfunction. These items are rated in terms of the frequency or severity of the problem on a four-point Likert scale (e.g., 0 = Not during the past month, 1 = Less than once a week, 2 = Once or twice a week, 3 = Three or more times a week). The sum of the component scores yields a global PSQI score that ranges from 0 to 21, with higher scores representing lower sleep quality.

Since there is no consensus on the PSQI threshold for longevous persons, we used the 7/8 global PSQI score as a cutoff of sleep disorder rather than the original 4/5 cutoff recommended by the tool developers. A cut-off score of >7 point was confirmed more appropriate to determine poor sleep in clinical practice (38–40). If any item under a component had a missing value, the component was defined as missing; 42 participants (4.4%) had at least one missing value for seven components. We performed multiple imputation to address the missing values (41).

#### The 15-Item Geriatric Depression Scale

Depression symptoms were measured using the Chinese version short form geriatric depression scale (GDS-15), which consists of 15 dichotomous items assessing the presence of depression symptoms during the last week (42). The responses to the 15 dichotomous items of this scale were scored and higher scores indicate more depressive symptoms (possible range 0–15; observed range of 0–15). The GDS-15 has been proven to be useful for assessing depressive symptoms among very old people (43, 44).

#### The Barthel Index for Activities of Daily Living

The Barthel Index consists of 10 items that measures a person’s physical activities of daily living (ADL) and is commonly used as a proxy for physical function (45). Daily activities measured by the Barthel Index include grooming, feeding, dressing, bathing, toilet use, transferring from bed to chair, walking, stair climbing, bowel continence, and urinary continence. Each item of the ADL is rated on a scale with a given number of points assigned to each level of activity, and the total score ranges from 0 to 100 points with five-point increments. A higher score indicates higher levels of physical function. Items of the Barthel index are regarded as dependent if they are performed with any help from other people. In our previous study, the functional dependence of ADL of centenarians was 71.2% (46).

#### The Satisfaction With Life Scale

The Satisfaction With Life Scale (SWLS) is a short five-item instrument and was developed as a way to assess an individual’s cognitive judgment of their satisfaction with their life as a whole. Participants completing the questionnaire are asked to judge how they feel about each of the statements using a seven-point Likert scoring system, with 1 being “strongly disagree” with the statement and 7 being “strongly agree” with the statement. Scale scores range from 5 to 35, with higher scores indicating greater life satisfaction (47).

#### Visual Analog Scales

Visual analog scales (VAS) are psychometric response scales used to measure subjective characteristics or attitudes and have been used in the past for a multitude of disorders. The VAS score records an individual’s self-rated health on a 20-cm, vertical visual analog scale ranging from 0 to 100, with notes at both ends labeling “the worst health you can imagine” (at 0) and “the best health you can imagine” (at 100). In our previous study, the mean VAS score was 66.9 among oldest-old populations and the respondents who had good sleeping quality reported higher VAS score by 7.09 on average, compared to those who had poor sleeping quality (48).

### Statistical Analysis

The 958 participants were randomly assigned into two subsamples. Normal continuous variables were described as the mean ± SD; skewed continuous variables were described as median with upper and lower quartiles; categorical variables were described as numbers with percentages. For statistical analysis, continuous variables were compared using Student’s t test (normal distribution) or Wilcoxon rank-sum test (skewed distribution); categorical variables were compared using Chi-squared test. As several variables were ordinal, we used Spearman correlation coefficient to measure the correlations between global PSQI score and each of its components, the GDS-15, the ADL, the SWLS, and the VAS. Furthermore, the Cronbach’s alpha coefficient (49) was used to measure the internal consistency of the seven components with the global score of the original PSQI and revised scales (after certain items were deleted). A Cronbach’s α coefficient greater than 0.7 indicates good internal consistency (50).

Parallel analyses (PA) (51) were performed to determine factor numbers with 500 random data matrices. The principal component analysis (PCA) and maximum likelihood (ML) extraction methods are the most widely used in studies conducting an EFA of the PSQI components (26, 31, 52, 53). We simultaneously used the above two extraction methods to test the stability of loadings as results may differ with variation in methodology. Given that some components may cross between different factors (especially component 1 sleep quality), an oblique rotation was performed to redistribute factor loadings which allows for correlations between factors. The criteria for factor extraction included: (1) eigenvalues >1, (2) loadings of each item ≥ 0.4, and (3) factors from the real data with eigenvalues greater than the corresponding eigenvalue from the random data (either the average or the 95th percentile) were retained in PA. Factor loadings were using against the following criteria: ≥0.71 (excellent), 0.63–0.70 (very good), 0.55–0.62 (good), 0.45–0.54 (fair), 0.32–0.44 (poor), and<0.32 (unacceptable and deleted from factor) (54). Given that the multivariate normality assumption was not met in our dataset and the PSQI scores were ordinal, a confirmatory factor analysis using the weighted least squares (WLS) method was conducted in the validation set using Mplus Version 7.4. Both absolute fit indexes (χ^2^/*df*; root mean square error of approximation, RMSEA) and value-added fit indexes (comparative fit index, CFI; normed fit index, NFI; incremental fit index, IFI; Tucker-Lewis index, TLI; relative fit index, RFI) were used. A nonsignificant χ^2^ statistic indicates that the fit of the restricted model is similar to that of the unrestricted model. However, the χ^2^ statistic is sensitive to small deviations in model fit with large samples (55). For well-specified models, a χ^2^/*df* of 5 or less and an RMSEA of 0.1 or less reflect an acceptable fit. For the CFI, NFI, IFI, TLI and RFI, values greater than 0.90 are typically considered acceptable. We also conducted subgroup analyses for the alternative model across demographic characteristics (gender, education, residence, etc.) to examine the stability of the model. Statistical significance was accepted at the two-sided 0.05 level and confidence interval was computed at the 95% level. Statistical analyses were performed with Statistic Package for Social Science (SPSS) version 22.0 and Mplus Version 7.4.

## Results

A total of 958 centenarians (*M* = 102.8 years old, *SD* = 2.7) were investigated with the Chinese version of the PSQI scale. The majority of participants were female (81.8%), Han (88.4%), illiterate (91.1%), rural residents (65.7%), lived with families (89.9%), and widowed or divorced (89.9%). The mean ± SD of the global PSQI scores was 8.44 ± 3.09, and the overall prevalence rate of poor sleep quality was 58.25% (cutoff=7/8). The characteristics of the total sample as well as the training/validation set are described in **Table 1**. After randomization, no significant differences were found across demographic characteristics between the two subsamples.

**Table 1:** Characteristics of 958 participants according to two subsamples.

Characteristics	Total sample(*N*=958)	Training set(*N*=479)	Validation set(*N*=479)	*P*-value
PSQI-7 score	8.44 ± 3.09	8.27 ± 3.08	8.61 ± 3.11	0.197^a^
Age, mean ± SD	102.79 ± 2.72	102.84 ± 2.89	102.76 ± 2.53	0.660^b^
BMI, mean ± SD	18.94 ± 3.63	18.86 ± 3.58	19.06 ± 3.68	0.453^a^
Female, n (%)	784(81.84%)	398(83.09%)	386(80.58%)	0.315^c^
Han ethnic, n (%)	847(88.41%)	422(88.10%)	425(88.73%)	0.762^c^
Illiterate, n (%)	873(91.13%)	441(92.07%)	432(90.19%)	0.364^c^
Rural, n (%)	629(65.66%)	314(65.55%)	315(65.76%)	0.954^c^
Divorced or widowed, n (%)	861(89.87%)	428(89.35%)	433(90.39%)	0.669^c^
Living with families, n (%)	822(85.80%)	414(86.43%)	408(85.18%)	0.172^c^
SWLS score	21.80 ± 7.25	22.25 ± 7.03	21.35 ± 7.44	0.025^b^
ADL score	74.69 ± 25.19	75.16 ± 25.09	74.23 ± 25.32	0.436^b^
GDS-15 score	5.28 ± 3.14	5.21 ± 3.09	5.37 ± 3.19	0.459^b^
VAS score	60.22 ± 17.94	61.39 ± 17.19	59.05 ± 18.62	0.044^b^

SD, standard deviation; BMI, body mass index; SWLS, Satisfaction with Life Scale; ADL, activity of daily living; VAS, Visual Analog Scale; GDS-15, the 15-item geriatric depression scale; PSQI, Pittsburgh Sleep Quality Index.

a Student’s t test.

b Wilcoxon rank-sum test.

c Chi-squared test.

**Figure 3:**
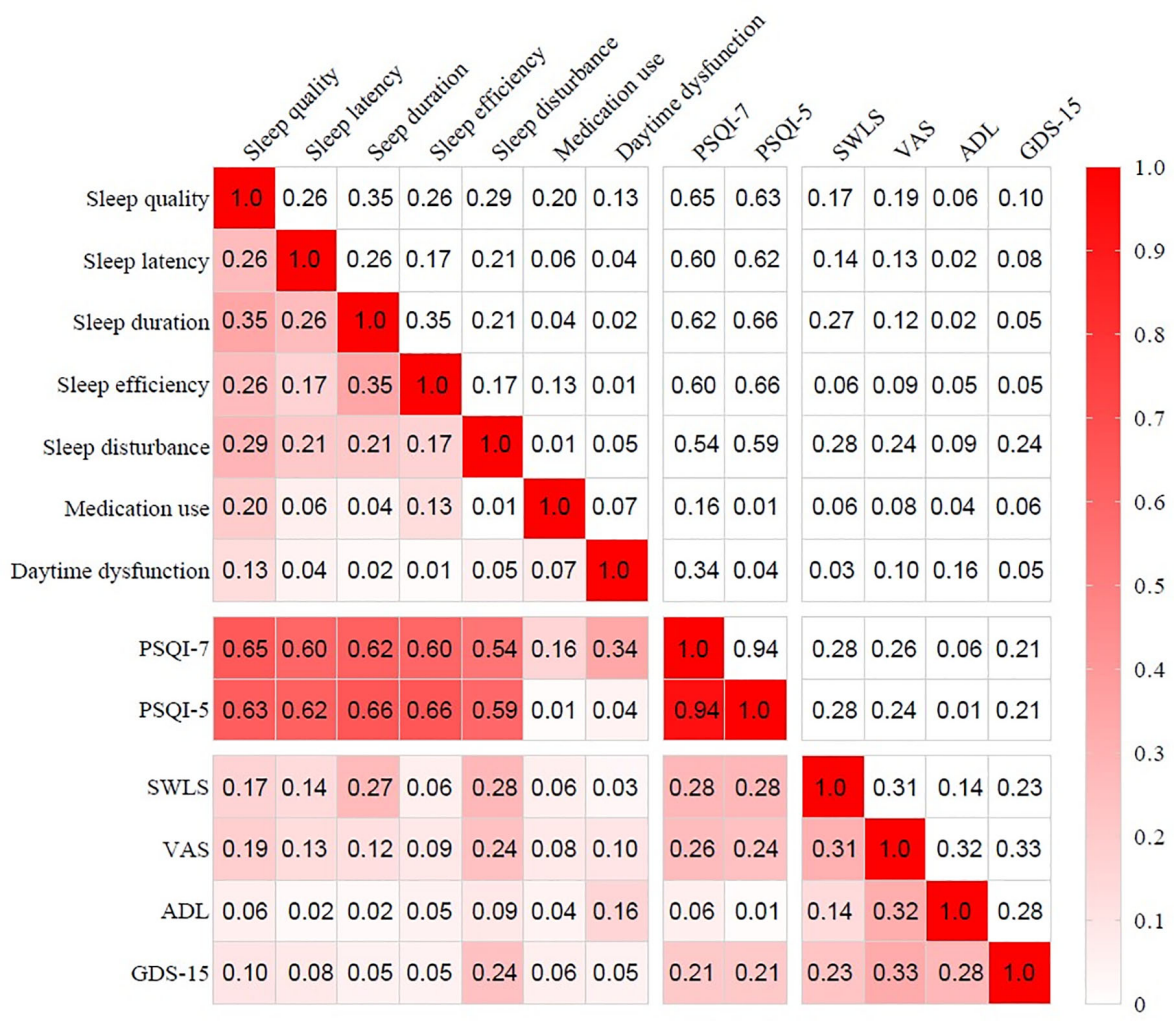
Correlation coefficients matrix of PSQI with each component, SWLS, VAS, ADL and GDS-15. PSQI, Pittsburgh sleep quality index; ADL, activity of daily living; VAS, Visual Analog Scale; GDS-15, 15-item of geriatric depression scale; SWLS, satisfaction with life scale.

As **Figure 3** shows, the global PSQI score was significantly correlated with each component (*P*<0.01). Among the seven components, the correlations between the global PSQI score and the medication use (*r*=0.136) and daytime dysfunction (*r*=0.315) components were relatively low, and these components were not significantly correlated with some other components. The participants’ responses to each component are shown in **Table 2**. The Cronbach’s alpha coefficient of the global PSQI was 0.68, and it increased to 0.78 after two components (medication use and daytime dysfunction) were deleted.

**Table 2:** Participants’ response to Pittsburgh sleep quality index (PSQI) and reliability of the scale (n=958).

Content	Component score (N=958)							Cronbach’s α coefficient^1^		
	0	1	2	3	Missing ratio	Mean ± SD	Median (Q25, Q75)	Total sample *N*=958	Training set *N*=479	Validation set *N*=479
Component 1 sleep quality	28 (2.92%)	277 (28.91)	528 (55.11)	125 (13.05%)	0	1.78 ± 0.70	2 (1,2)	0.561	0.575	0.544
Component 2 sleep latency	171 (17.85%)	145 (15.14)	404 (41.46%)	241 (25.16%)	22 (2.3%)	1.74 ± 1.03	2 (1,3)	0.608	0.609	0.605
Component 3 sleep duration	675 (70.46%)	175 (18.27%)	58 (6.05%)	50 (5.22%)	2 (0.2%)	0.46 ± 0.83	0 (0,1)	0.577	0.586	0.567
Component 4 sleep efficiency	212 (22.13%)	276 (28.81%)	233 (24.32%)	237 (24.74%)	11 (1.1)	1.52 ± 1.09	1 (1,2)	0.624	0.641	0.605
Component 5 sleep disturbance	77 (8.04%)	291 (30.38%)	328 (34.24%)	262 (27.35%)	4 (0.4%)	1.81 ± 0.93	2 (1,3)	0.632	0.641	0.623
Component 6 medication use	934 (97.50%)	5 (0.52%)	3 (0.31%)	16 (1.67%)	40 (4.2%)	0.06 ± 0.41	0 (0,0)	0.724	0.737	0.711
Component 7 daytime dysfunction	321 (33.51%)	301 (31.42%)	287 (29.95%)	49 (5.12%)	8 (0.8%)	1.07 ± 0.91	1 (0,2)	0.694	0.705	0.683
PSQI-7						8.44 ± 3.09	8 (6,10)	0.675	0.685	0.663
PSQI-5 (delete component 6 and 7)						7.31 ± 2.88	7 (5,9)	0.778	0.781	0.775

^1^Cronbach’s α coefficient of the scale when each item was deleted.

**Figure 4:**
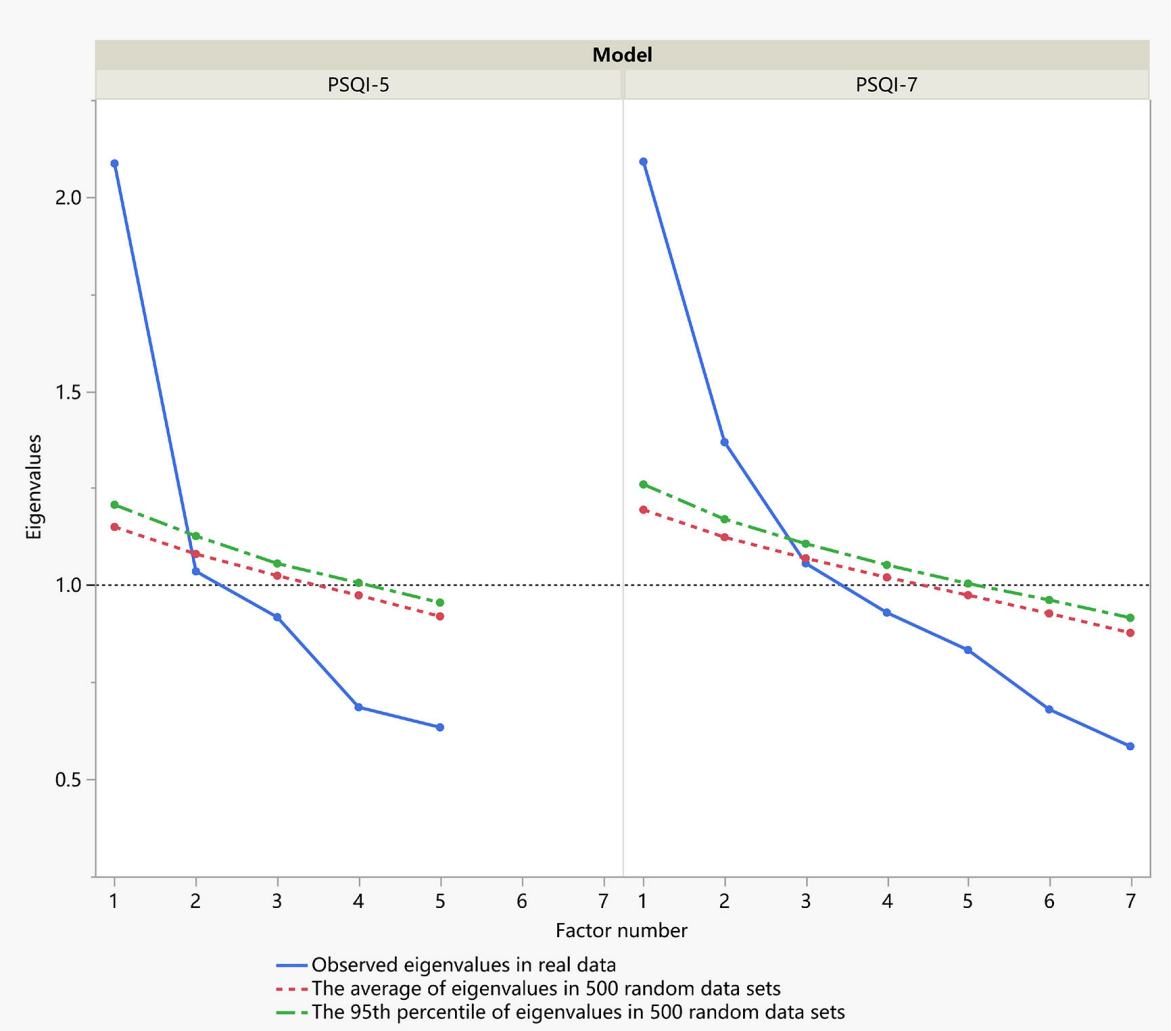
Scree plots of actual versus randomly generated eigenvalues in parallel analysis. Eigenvalues 1 and greater than the corresponding eigenvalue from the random data (either the average or the 95th percentile) were retained.

Kaiser-Meyer-Olkin (*KMO*=0.78) and Bartlett’s sphere tests (χ^2^ = 328.98, *df*=21, *P*<0.001) supported the suitability of the data for factor analysis. An exploratory factor analysis with oblique rotation was performed in the training set. As **Figure 4** shows, parallel analysis confirmed two factors for components 1-7 and one factor for components 1–5. According to the PCA, two factors (factor 1 with components 1–5; factor 2 with components 6 and 7) were extracted with a total variance contribution rate of 46.9%. When components 6 and 7 were deleted, one factor (components 1–5) was extracted, which accounted for 43.6% of the variance. All five factor loadings are reasonable according to the given criteria. Each component’s loading with the PCA and ML methods is shown in **Table 3**. The results of factor loadings using the two extraction methods were basically consistent.

**Table 3:** Factor loadings of 7 components comparing two extraction methods.

Model Extraction	PSQI-7				PSQI-5	
	PCA		ML		PCA	ML
Number of Factors	1	2	2	1	1	1
Component-1	0.682^b^	0.385	0.721^a^	0.225	0.712^a^	0.593^c^
Component-2	0.606^c^	0.138	0.398^e^	−0.018	0.635^b^	0.556^c^
Component-3	0.751^a^	−0.169	0.561^c^	−0.228	0.741^a^	0.608^c^
Component-4	0.623^c^	−0.353	0.460^d^	−0.352	0.596^c^	0.478^d^
Component-5	0.541^d^	0.123	0.397^e^	−0.040	0.553^c^	0.455^d^
Component-6	−0.012	0.816^a^	0.118	0.604^c^		
Component-7	0.060	0.446^d^	0.099	0.386^e^		

Loadings are a = excellent, b = very good, c = good, d = fair, e = poor.

PSQI, Pittsburgh sleep quality index; PCA, principal component analysis; ML, maximum likelihood.

**Figure 5:**
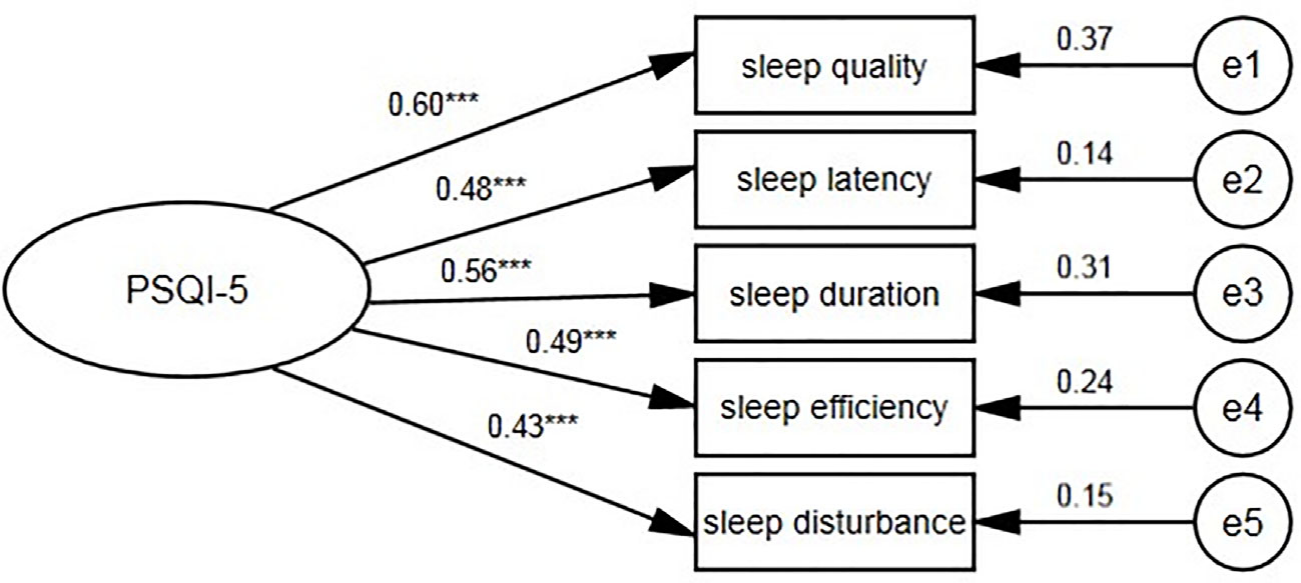
One-factor PSQI-5 model for the centenarians. PSQI, Pittsburgh sleep quality index. ^***^P < 0.001.

Confirmatory factor analyses were adopted for the validation set to compare the suitability of seven competition models. Model A was the original unidimensional structure with seven components, model B was a two-factor structure and model C was a one-factor model with five components. We also examined four commonly used PSQI models from previous studies examining elderly people (25, 28, 34, 52, 53). Details of the seven competing models are presented in **Table 4**. Some models were modified by theoretical residual correlations based on the modification index (≥4). To systematically compare the performance of all models, we used the following methods: (1) model fit indices; (2) factor loadings in the CFA model; (3) correlation with the original global PSQI score ; and (4) Cronbach’s α coefficient. The one-factor model C (**Figure 5**) with five components (χ^2^/*df* =1.59, *P*=0.157, CFI=0.99, RMSEA=0.03) fit the data better than other models, and no MI indicated model modification. To further explore the stability of model C, subgroup analyses (**Table 5**) were performed in the validation set. No significant difference was found across demographic characteristics based on _Δ_χ^2^ (*P*>0.05). Moreover, this revised PSQI-5 model fit both the total sample (χ^2^/*df* =2.66, *P*=0.080, CFI=0.97, RMSEA= 0.04) and the training set (χ^2^/*df* =2.98, *P*=0.010, CFI=0.96, RMSEA= 0.06) in a satisfactory way.

**Table 4:** Comparison of seven competing Pittsburgh sleep quality index (PSQI) models for the validation set.

Model	Factor/item	χ^2^/*df*	P	RMSE A90%CI	NFI	RFI	IFI	TLI	CFI	Factor loadings average (min, max)	Correlationwith PSQI	Cronbach’s α coefficient
A	1/7	2.12	0.016	0.05(0.02–0.08)	0.92	0.85	0.96	0.91	0.95	0.37(0.03,0.60)	1.000	0.675
B	2/7	4.87	<0.001	0.09(0.07–0.11)	0.79	0.65	0.83	0.69	0.83	0.42(0.23,0.73)	1.000	0.675
**C**	**1/5**	**1.59**	**0.157**	**0.03(0.00–0.08)**	**0.96**	**0.93**	**0.99**	**0.97**	**0.99**	**0.51(0.43,0.60)**	**0.945**	**0.778**
D	3/6	3.18	<0.001	0.06(0.03–0.09)	0.87	0.88	0.90	0.93	0.90	0.46(0.22, 0.61)	0.958	0.724
E	1/6	2.11	0.032	0.04(0.00–0.05)	0.93	0.87	0.96	0.93	0.96	0.41(0.02,0.61)	0.958	0.724
F	3/7	4.36	<0.001	0.08(0.07–0.10)	0.87	0.81	0.90	0.85	0.90	0.42(0.07, 0.63)	1.000	0.675
G	2/6	5.53	<0.001	0.08(0.06–0.11)	0.72	0.76	0.76	0.82	0.762	0.47(0.12, 0.64)	0.958	0.724

RMSEA, root mean square error of approximation; NFI, normed fit index; RFI, relative fit index; IFI, incremental fit index; TLI, Tucker-Lewis index; CFI, comparative fit index; CI, confidence interval.

Model A: one factor with components 1–7.

Model B: factor 1 (with component 1, 2, 3, 4, 5); factor 2 (with component 6, 7).

Model C: one factor with component 1–5.

Model D: factor 1 (with component 1, 2); factor 2 (with component 3, 4); factor 3 (with component 5, 7).

Model E: factor 1 (with component 1, 2, 3, 4, 5, 7).

Model F: factor 1 (with component 1, 2); factor 2 (with component 3, 4); factor 3 (with component 5, 6, 7).

Model G: factor 1 (with component 1, 2, 3, 4); factor 2 (with component 5,7)

The bold content emphasizes the final accepted model C in multiple CFA.

**Table 5:** Multigroup configural invariance analysis of PSQI-5 model for the validation set.

Characteristics	_Δ_χ^2^	*df*	*P*-value	_Δ_CFI	_Δ_NFI	_Δ_RMSEA
Gender	0.528	4	0.971	0.023	0.013	0.003
Education	5.004	4	0.287	0.023	0.021	0.004
Residence	1.726	4	0.786	0.045	0.030	0.006
Marriage status	5.576	4	0.212	0.033	0.046	0.004
Living status	3.766	4	0.439	0.051	0.013	0.003

CFI, comparative fit index; NFI, normed fit index; RMSEA, root mean square error of approximation, PSQI, Pittsburgh sleep quality index.

Spearman correlations were significant among GDS-15, SWLS, VAS, and PSQI, the magnitudes of correlations were presented in **Figure 2**. The global PSQI score was significantly correlated with summed scores of GDS-15(*r*=0.215, *P*<0.01), SWLS (*r*=0.287, *P*<0.01) and VAS (*r*=0.264, *P*<0.01). However, the correlation between PSQI and ADL was not statistically significant (*r*=-0.063, *P*=0.065).

## Discussion

To the best of our knowledge, this is the first study to date to examine the psychometric properties of the Chinese language version of the PSQI in a community sample of centenarians with a large sample size. The core finding was that the original PSQI-7 had adequate internal consistency and factor stability, while the revised PSQI-5 had better internal consistency and factor stability as an instrument to screen centenarians’ sleep quality.

**Figure 6:**
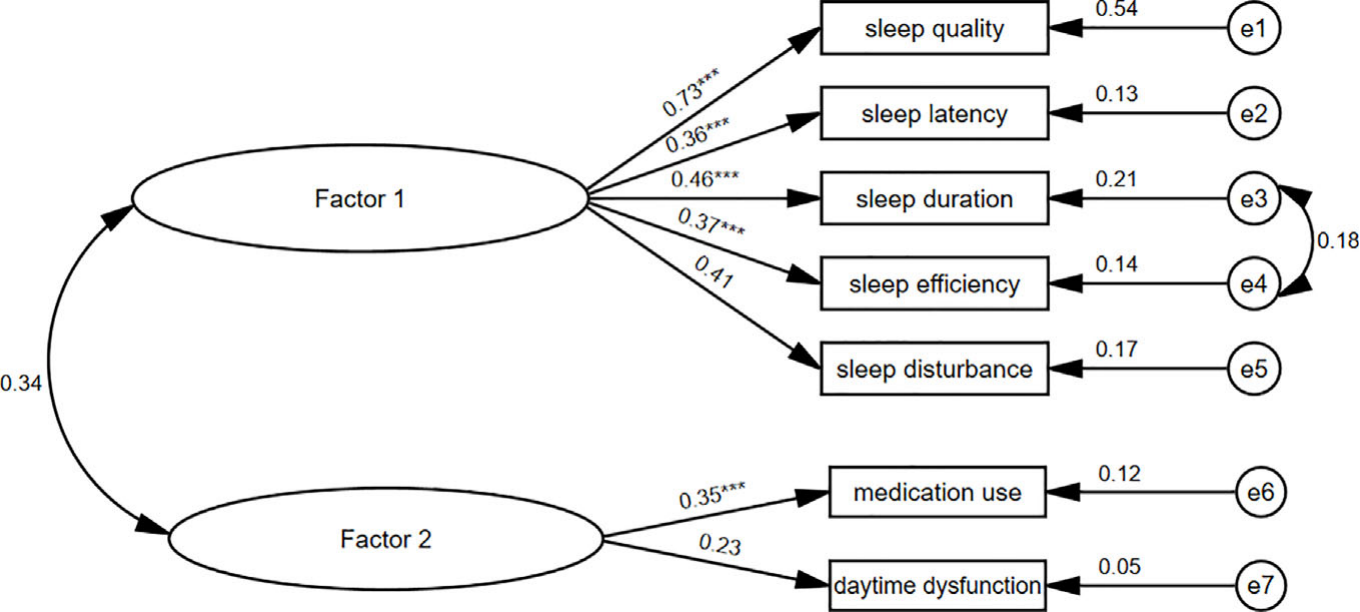
Two-factor PSQI-7 model for the centenarians. PSQI, Pittsburgh sleep quality index. ^***^P < 0.001.

The PSQI showed a fair Cronbach’s α coefficient and adequate variance contribution rate compared with previous aged population-based studies from Portugal (25), the United States (53) and China (34). However, two components (medication use and daytime dysfunction) strongly influenced the reliability and construct validation of the scale. The elevated Cronbach’s α coefficient (from 0.68 to 0.78) after deleting the two components suggested the limited contributions of the two deleted components. Besides, as **Figure 3** showed, the two deleted components had poor item-total correlations with the global PSQI score (*r*<0.4) as well as other reserved components (*r*<0.2). Their factor loadings were unacceptable in both one-factor and two-factor CFA models (<0.4). We obtained a one-factor model with good loadings from PCA on the remaining five components. In the fitness comparison of the seven competing models shown in **Table 5**, neither the original unidimensional model A nor the two-factor model B (**Figure 6**) showed acceptable goodness of fit for the validation set. Since the EFA is a data-driven approach that may lead to spurious deviations from well-known factor structures, we added four commonly used PSQI models for comparison, but none of them fit the data well. The revised one-factor PSQI-5 model C fit the data well with a non-significant χ^2^/*df* (*P*=0.157), and had good performance across demographic characteristics. Therefore, the one-factor PSQI-5 can be recommended as the best model for community-dwelling Chinese centenarians. One of PSQI validation studies likewise indicated factorial validity of a unidimensional scale with the same five components among women aged ≥70 in America (56). While another study has shown validity of a three-component unidimensional PSQI scale (sleep quality, sleep latency and sleep disturbances) in a sample of community-dwelling older Malaysians (57). These previous similar results support the feasibility of simplifying PSQI components in clinical practice.

Discordance with the medication use component was common in previous studies with either clinical or nonclinical samples. Several published findings showed poor correlations or small factor loading (22, 25, 33, 34, 53, 58–60) for this component in the PSQI framework. The medication use component was also removed in a previous study to improve the model fitness in a Chinese clinical setting (34). Another study about community-dwelling older adults from Portugal removed this component (25). Centenarians in this study are more likely to be illiterate and lacks medical service accessibility. Their awareness of sleep problems and their use of sleep medications were relatively low: only 1.98% reported regular use of sleep medications. It is noted that removing medication use component was based on psychometrical method. However, there are some situations in which information on medication use may justify its inclusion. In other words, the best possible psychometrics are not always the highest consideration. Also, the use and availability of medication may differ substantially among populations and regions. Therefore, our findings may not generalize to other samples of centenarians from other regions. Although 35.07% of the centenarians showed serious daytime dysfunction, this component was not consistent with the others. Similar results were found in older men (61) and women (56) with osteoporotic fractures and in a study on black and white octogenarians (56). Centenarians in our study are tend to be became the crookback and often companied with chronic pain such as arthritis, those symptoms associated with poor sleep quality but not daytime dysfunction; and sometime sitting position might relieve the pain from lying position. The centenarians’ sleep quality was not significantly correlated with physical function (Barthel index of ADL). Although daytime dysfunction often accompanies inadequate nighttime sleep (62), the association was not common in older people with physical function impairments.

The dimensionality of the PSQI is highly controversial. Several studies suggested the original unidimensional measure (34, 57, 63, 64), while others supported the multidimensional index (two or three factors) (25, 53, 65, 66). Diverse sample characteristics and nonuniform methodologies (e.g., factor rotation and extraction methods; estimation method selection) may account for the conflicting results. Groups observed in previous studies have highly heterogeneous demographic characteristics and health status. In addition, the results of single confirmatory and exploratory factor analyses may be inconsistent, as shown in several studies (22, 25, 67). Thus, it is appropriate to refer the results of other studies directly. Using across-validation approach, we confirmed the one-factor PSQI-5 model as the optimal structure in our sample (22, 53).

Significant correlations between the global PSQI score and multiple self-reported health outcomes provided evidence that the Chinese PSQI had appropriate divergent validity in centenarians. This finding is consistent with correlations obtained in samples of the elderly (68, 69). Considering the potential loss of information by deleting two components, we examined the screening consistency of the two PSQI scales. When the sleep quality cutoff was 7/8 for the original PSQI-7, we observed a satisfactory Kappa coefficient (0.801, 95% CI: 0.736–0.863) with the revised PSQI-5(cutoff=6/7). This indicated a high consistency of screening ability between the two scales; however, their screening abilities need to be tested against a standard clinical diagnosis. The PSQI-5 has appropriate reliability and validity compared to the original PSQI, and it may be useful in clinical practice because it may slightly reduce the time needed for calculating scores. In addition, our results supported the unidimensional formulation of the PSQI suggested by Buysse et al. (17), which makes it feasible to use the global score rather than separate factor scores, as a global score is often sufficient for screening purposes (70).

Some limitations of the current study should be acknowledged. First, the sample was representative of centenarians residing in low- or middle-income regions; extrapolation to centenarians from other settings should be performed with caution. Second, as the current household registration system did not exist in China sixty years ago, the age of the participants based on the Chinese identity card might not be well-validated because of a lack of birth certificates. Nevertheless, strict quality control with three-step age verification methods has been taken to avoid age inaccuracy, which attenuates age exaggeration. Third, this study was conducted in a community setting and did not include varied diagnostic groups (e.g., sleep apnea, and insomnia). Thus, test attributes such as sensitivity, specificity, and known-group validity could not be evaluated. These attributes should be measured to further validate the PSQI in this age group.

In conclusion, the current findings validate the original PSQI-7, and demonstrate that the revised PSQI-5 had a satisfactory univariate factor structure for assessing centenarians’ sleep in this sample. Future studies should validate the factor structure and psychometric properties of PSQI and determine an appropriate cutoff score to identify sleep quality in community-based centenarians from varied backgrounds.

## Data Availability Statement

The raw data supporting the conclusions of this article will be made available by the authors, without undue reservation.

## Ethics Statement

The studies involving human participants were reviewed and approved by: The ethics committee of the Hainan branch of the Chinese People’s Liberation Army General Hospital (Sanya, Hainan) approved the study protocol (no. of serial: 301hn11201601). The patients/participants provided their written informed consent to participate in this study. Written informed consent was obtained from the individual(s) for the publication of any potentially identifiable images or data included in this article.

## Author Contributions

CZ, YZ, and YY proposed the concept and design, analyzed and interpreted the data, and wrote the manuscript; CZ, HZ, and YY interpreted the data, drafted and edited the manuscript, supervised the study, and obtained funding; MZ, ZL, CC, and DB drafted and edited the manuscript. All authors contributed to the article and approved the submitted version. YY and YZ are guarantors.

## Funding

The research was funded by the National Key R&D Program of China (2018YFC2000400), National Natural Sciences Foundation of China (71490732, 81903392, 81941021), and China Postdoctoral Science Foundation funded project (2019M650359).

## Conflict of Interest

The authors declare that the research was conducted in the absence of any commercial or financial relationships that could be construed as a potential conflict of interest.

## References

[1] Reid KJ Martinovich Z Finkel S Statsinger J Golden R Harter K Sleep: a marker of physical and mental health in the elderly. Am J Geriatr Psychiatry (2006) 14:860–6. 10.1097/01.JGP.0000206164.56404.ba 17001025

[2] Helbig AK Doring A Heier M Emeny RT Zimmermann AK Autenrieth CS Association between sleep disturbances and falls among the elderly: results from the German Cooperative Health Research in the Region of Augsburg-Age study. Sleep Med (2013) 14:1356–63. 10.1016/j.sleep.2013.09.004 24157099

[3] Stone KL Ewing SK Lui LY Ensrud KE Ancoli-Israel S Bauer DC Self-reported sleep and nap habits and risk of falls and fractures in older women: the study of osteoporotic fractures. J Am Geriatr Soc (2006) 54:1177–83. 10.1111/j.1532-5415.2006.00818.x 16913982

[4] Gildner TE Liebert MA Kowal P Chatterji S Josh Snodgrass J Sleep duration, sleep quality, and obesity risk among older adults from six middle-income countries: findings from the study on global AGEing and adult health (SAGE). Am J Hum Biol (2014) 26:803–12. 10.1002/ajhb.22603 25130760

[5] Blackwell T Yaffe K Ancoli-Israel S Schneider JL Cauley JA Hillier TA Poor sleep is associated with impaired cognitive function in older women: the study of osteoporotic fractures. J Gerontol A Biol Sci Med Sci (2006) 61:405–10. 10.1093/gerona/61.4.405 16611709

[6] Haseli-Mashhadi N Dadd T Pan A Yu Z Lin X Franco OH Sleep quality in middle-aged and elderly Chinese: distribution, associated factors and associations with cardio-metabolic risk factors. BMC Public Health (2009) 9:130. 10.1186/1471-2458-9-130 19426521 PMC2683822

[7] Suzuki E Yorifuji T Ueshima K Takao S Sugiyama M Ohta T Sleep duration, sleep quality and cardiovascular disease mortality among the elderly: a population-based cohort study. Prev Med (2009) 49:135–41. 10.1016/j.ypmed.2009.06.016 19573557

[8] Soldatos CR Paparrigopoulos TJ Sleep physiology and pathology: pertinence to psychiatry. Int Rev Psychiatry (2005) 17:213–28. 10.1080/09540260500104565 16194793

[9] Barthlen GM Stacy C Dyssomnias, parasomnias, and sleep disorders associated with medical and psychiatric diseases. Mt Sinai J Med (1994) 61:139–59. 8022426

[10] Woodward M Sleep in older people. Rev Clin Gerontol (2012) 22:130–49. 10.1017/S0959259811000232

[11] Nations By United Department of Economic and Social Affairs; World Population Ageing 2009. Population Dev Rev (2011) 37:403. 10.1111/j.1728-4457.2011.00421.x

[12] Andersen-Ranberg K Schroll M Jeune B Healthy centenarians do not exist, but autonomous centenarians do: a population-based study of morbidity among Danish centenarians. J Am Geriatr Soc (2001) 49:900–8. 10.1046/j.1532-5415.2001.49180.x 11527481

[13] Tafaro L Cicconetti P Baratta A Brukner N Ettorre E Marigliano V Sleep quality of centenarians: cognitive and survival implications. Arch Gerontol Geriatr (2007) 44(Suppl 1):385–9. 10.1016/j.archger.2007.01.054 17317480

[14] Jirong Y Changquan H Hongmei W Bi-Rong D Association of sleep quality and dementia among long-lived Chinese older adults. Age (Dordr) (2013) 35:1423–32. 10.1007/s11357-012-9432-8 PMC3705112 22669593

[15] Gu D Sautter J Pipkin R Zeng Y Sociodemographic and health correlates of sleep quality and duration among very old Chinese. Sleep (2010) 33:601–10. 10.1093/sleep/33.5.601 PMC2864875 20469802

[16] Klein L Gao T Barzilai N Milman S Association between Sleep Patterns and Health in Families with Exceptional Longevity. Front Med (Lausanne) (2017) 4:214. 10.3389/fmed.2017.00214 29276708 PMC5727046

[17] Buysse DJ Reynolds CF,3 Monk TH Berman SR Kupfer DJ The Pittsburgh Sleep Quality Index: a new instrument for psychiatric practice and research. Psychiatry Res (1989) 28:193–213. 10.1016/0165-1781(89)90047-4 2748771

[18] Sowers MF Zheng H Kravitz HM Matthews K Bromberger JT Gold EB Sex steroid hormone profiles are related to sleep measures from polysomnography and the Pittsburgh Sleep Quality Index. Sleep (2008) 31:1339–49. PMC2572739 18853931

[19] Mollayeva T Thurairajah P Burton K Mollayeva S Shapiro CM Colantonio A The Pittsburgh sleep quality index as a screening tool for sleep dysfunction in clinical and non-clinical samples: A systematic review and meta-analysis. Sleep Med Rev (2016) 25:52–73. 10.1016/j.smrv.2015.01.009 26163057

[20] Ji-Rong Y Hui W Chang-Quan H Bi-Rong D Association between sleep quality and arterial blood pressure among Chinese nonagenarians/centenarians. Med Sci Monit (2012) 18:Ph36–42. 10.12659/msm.882512 22367137 PMC3560755

[21] Yan Z Chang-Quan H Zhen-Chan L Bi-Rong D Association between sleep quality and body mass index among Chinese nonagenarians/centenarians. Age (Dordr) (2012) 34:527–37. 10.1007/s11357-011-9251-3 PMC3337939 21590342

[22] Magee CA Caputi P Iverson DC Huang XF An investigation of the dimensionality of the Pittsburgh Sleep Quality Index in Australian adults. Sleep Biol Rhythms (2008) 6:222–7. 10.1111/j.1479-8425.2008.00371.x

[23] Anandakumar D Dayabandara M Ratnatunga SS Hanwella R de Silva VA Validation of the Sinhala version of the Pittsburgh Sleep Quality Index. Ceylon Med J (2016) 61:22–5. 10.4038/cmj.v61i1.8255 27031975

[24] Chong Alice ML Cheung C-K Factor structure of a Cantonese-version Pittsburgh Sleep Quality Index. Sleep Biol Rhythms (2012) 10:118–25. 10.1111/j.1479-8425.2011.00532.x

[25] Becker NB de Neves Jesus S Adaptation of a 3-factor model for the Pittsburgh Sleep Quality Index in Portuguese older adults. Psychiatry Res (2017) 251:298–303. 10.1016/j.psychres.2017.02.033 28236782

[26] Raniti MB Waloszek JM Schwartz O Allen NB Trinder J Factor structure and psychometric properties of the Pittsburgh Sleep Quality Index in community-based adolescents. Sleep (2018) 41:1–12. 10.1093/sleep/zsy066 29608755

[27] Tsai PS Wang SY Wang MY Su CT Yang TT Huang CJ Psychometric evaluation of the Chinese version of the Pittsburgh Sleep Quality Index (CPSQI) in primary insomnia and control subjects. Qual Life Res (2005) 14:1943–52. 10.1007/s11136-005-4346-x 16155782

[28] Sohn S II Kim DH Lee MY Cho YW The reliability and validity of the Korean version of the Pittsburgh Sleep Quality Index. Sleep Breath (2012) 16:803–12. 10.1007/s11325-011-0579-9 21901299

[29] Del Rio Joao KA Becker NB de Neves Jesus S Isabel Santos Martins R Validation of the Portuguese version of the Pittsburgh Sleep Quality Index (PSQI-PT). Psychiatry Res (2017) 247:225–9. 10.1016/j.psychres.2016.11.042 27923147

[30] Kotronoulas GC Papadopoulou CN Papapetrou A Patiraki E Psychometric evaluation and feasibility of the Greek Pittsburgh Sleep Quality Index (GR-PSQI) in patients with cancer receiving chemotherapy. Supp Care Cancer (2011) 19:1831–40. 10.1007/s00520-010-1025-4 20972588

[31] Manzar MD BaHammam AS Hameed UA Spence DW Pandi-Perumal SR Moscovitch A Dimensionality of the Pittsburgh Sleep Quality Index: a systematic review. Health Qual Life Outcomes (2018) 16:89. 10.1186/s12955-018-0915-x 29743066 PMC5944037

[32] Manzar D Zannat W Moiz JA Spence DW Hussain ME Factor scoring models of the Pittsburgh Sleep Quality Index: a comparative confirmatory factor analysis. Biol Rhythm Res (2016) 47:1–28. 10.1080/09291016.2016.1202375

[33] Fontes F Goncalves M Maia S Pereira S Severo M Lunet N Reliability and validity of the Pittsburgh Sleep Quality Index in breast cancer patients. Supp Care Cancer (2017) 25:3059–66. 10.1007/s00520-017-3713-9 28455545

[34] Zhu B Xie M Park CG Kapella MC Adaptation of the Pittsburgh Sleep Quality Index in Chinese adults with type 2 diabetes. J Chin Med Assoc (2018) 81:242–7. 10.1016/j.jcma.2017.06.021 PMC6873698 29258729

[35] Morris JL Rohay J Chasens ER Sex Differences in the Psychometric Properties of the Pittsburgh Sleep Quality Index. J Womens Health (Larchmt) (2018) 27:278–82. 10.1089/jwh.2017.6447 PMC5865255 29154713

[36] Wang L Li Y Li H Holdaway J Hao Z Wang W Regional aging and longevity characteristics in China. Arch Gerontol Geriatr (2016) 67:153–9. 10.1016/j.archger.2016.08.002 27544461

[37] He Y Zhao Y Yao Y Yang S Li J Liu M Cohort Profile: The China Hainan Centenarian Cohort Study (CHCCS). Int J Epidemiol (2018) 47:694–5h. 10.1093/ije/dyy017 29506028

[38] Fichtenberg NL Zafonte RD Putnam S Mann NR Millard AE Insomnia in a post-acute brain injury sample. Brain Inj (2002) 16:197–206. 10.1080/02699050110103940 11874613

[39] Beck SL Schwartz AL Towsley G Dudley W Barsevick A Psychometric evaluation of the Pittsburgh Sleep Quality Index in cancer patients. J Pain Symptom Manage (2004) 27:140–8. 10.1016/j.jpainsymman.2003.12.002 15157038

[40] Carpenter JS Andrykowski MA Psychometric evaluation of the Pittsburgh Sleep Quality Index. J Psychosom Res (1998) 45:5–13. 10.1016/s0022-3999(97)00298-5 9720850

[41] Schafer JL Graham JW Missing data: our view of the state of the art. Psychol Methods (2002) 7:147–77. 10.1037/1082-989X.7.2.147 12090408

[42] Sheikh J Geriatric depression scale (GDS) : recent evidence and development of a shorter version. Clin Gerontol (1986) 5:165–73. 10.1300/J018v05n01_09

[43] Yao Y Fu S Zhang H Li N Zhu Q Zhang F The prevalence of depressive symptoms in Chinese longevous persons and its correlation with vitamin D status. BMC Geriatr (2018) 18:198. 10.1186/s12877-018-0886-0 30157761 PMC6114877

[44] Conradsson M Rosendahl E Littbrand H Gustafson Y Olofsson B Lovheim H Usefulness of the Geriatric Depression Scale 15-item version among very old people with and without cognitive impairment. Aging Ment Health (2013) 17:638–45. 10.1080/13607863.2012.758231 PMC3701937 23339600

[45] Mahoney F II Barthel DW Functional evaluation : the Barthel Index. Md State Med J (1965) 14:61–5. 10.1037/t02366-000 14258950

[46] Yao Y Fu S Shi Q Zhang H Zhu Q Zhang F Prevalence of functional dependence in Chinese centenarians and its relationship with serum vitamin D status. Clin Interv Aging (2018) 13:2045–53. 10.2147/CIA.S182318 PMC6200073 30410320

[47] Corrigan JD Kolakowsky-Hayner S Wright J Bellon K Carufel P The Satisfaction With Life Scale. J Head Trauma Rehabil (2013) 28:489–91. 10.1097/htr.0000000000000004 24189288

[48] Chen C Liu GG Shi QL Sun Y Zhang H Wang MJ Health-Related Quality of Life and Associated Factors among Oldest-Old in China. J Nutr Health Aging (2020) 24:330–8. 10.1007/s12603-020-1327-2 PMC7064459 32115616

[49] Cronbach LJ Warrington WG Time-limit tests: estimating their reliability and degree of speeding. Psychometrika (1951) 16:167–88. 10.1007/bf02289113 14844557

[50] McHorney CA Tarlov AR Individual-patient monitoring in clinical practice: are available health status surveys adequate? Qual Life Res (1995) 4:293–307. 10.1007/bf01593882 7550178

[51] Horn JL A rationale and test for the number of factors in factor analysis. Psychometrika (1965) 30:179–85. 10.1007/bf02289447 14306381

[52] Mariman A Vogelaers D Hanoulle I Delesie L Tobback E Pevernagie D Validation of the three-factor model of the PSQI in a large sample of chronic fatigue syndrome (CFS) patients. J Psychosom Res (2012) 72:111–3. 10.1016/j.jpsychores.2011.11.004 22281451

[53] Cole JC Motivala SJ Buysse DJ Oxman MN Levin MJ Irwin MR Validation of a 3-factor scoring model for the Pittsburgh sleep quality index in older adults. Sleep (2006) 29:112–6. 10.1093/sleep/29.1.112 16453989

[54] Price B Comrey Andrew L Lee Howard B A First Course in Factor Analysis. Technometrics (1993) 35:453. 10.1080/00401706.1993.10485363

[55] Hu LT Evaluating model fit. Struct Equation Model Concepts Issues Appl (1995) 76–99.

[56] Beaudreau SA Spira AP Stewart A Kezirian EJ Lui LY Ensrud K Validation of the Pittsburgh Sleep Quality Index and the Epworth Sleepiness Scale in older black and white women. Sleep Med (2012) 13:36–42. 10.1016/j.sleep.2011.04.005 22033120 PMC3586742

[57] Yunus RM Wazid SW Hairi NN Choo WY Hairi FM Sooryanarayana R Association between elder abuse and poor sleep: A cross-sectional study among rural older Malaysians. PloS One (2017) 12:e0180222. 10.1371/journal.pone.0180222 28686603 PMC5501458

[58] Guo S Sun W Liu C Wu S Structural Validity of the Pittsburgh Sleep Quality Index in Chinese Undergraduate Students. Front Psychol (2016) 7:1126. 10.3389/fpsyg.2016.01126 27551270 PMC4976124

[59] Babson KA Blonigen DM Boden MT Drescher KD Bonn-Miller MO Sleep quality among U.S. military veterans with PTSD: a factor analysis and structural model of symptoms. J Trauma Stress (2012) 25:665–74. 10.1002/jts.21757 23225033

[60] Tomfohr LM Schweizer CA Dimsdale JE Loredo JS Psychometric characteristics of the Pittsburgh Sleep Quality Index in English speaking non-Hispanic whites and English and Spanish speaking Hispanics of Mexican descent. J Clin Sleep Med (2013) 9:61–6. 10.5664/jcsm.2342 PMC3525990 23319906

[61] Spira AP Beaudreau SA Stone KL Kezirian EJ Lui LY Redline S Reliability and validity of the Pittsburgh Sleep Quality Index and the Epworth Sleepiness Scale in older men. J Gerontol A Biol Sci Med Sci (2012) 67:433–9. 10.1093/gerona/glr172 PMC3309871 21934125

[62] Shelgikar AV Chervin R Approach to and evaluation of sleep disorders. Continuum (Minneap Minn) (2013) 19:32–49. 10.1212/01.CON.0000427214.00092.0f 23385693

[63] de la Vega R Tome-Pires C Sole E Racine M Castarlenas E Jensen MP The Pittsburgh Sleep Quality Index: Validity and factor structure in young people. Psychol Assess (2015) 27:e22–7. 10.1037/pas0000128 26653055

[64] Rener-Sitar K John MT Bandyopadhyay D Howell MJ Schiffman EL Exploration of dimensionality and psychometric properties of the Pittsburgh Sleep Quality Index in cases with temporomandibular disorders. Health Qual Life Outcomes (2014) 12:10. 10.10.1186/1477-7525-12-10 24443942 PMC3902412

[65] Qiu C Gelaye B Zhong QY Enquobahrie DA Frederick IO Williams MA Construct validity and factor structure of the Pittsburgh Sleep Quality Index among pregnant women in a Pacific-Northwest cohort. Sleep Breath (2016) 20:293–301. 10.1007/s11325-016-1313-4 26810497 PMC5010363

[66] Passos MH Silva HA Pitangui AC Oliveira VM Lima AS Araujo RC Reliability and validity of the Brazilian version of the Pittsburgh Sleep Quality Index in adolescents. J Pediatr (Rio J) (2017) 93:200–6. 10.1016/j.jped.2016.06.006 27520731

[67] Jimenez-Genchi A Monteverde-Maldonado E Nenclares-Portocarrero A Esquivel-Adame G de la Vega-Pacheco A Reliability and factorial analysis of the Spanish version of the Pittsburg Sleep Quality Index among psychiatric patients. Gac Med Mex (2008) 144:491–6. 19112721

[68] Grandner MA Kripke DF Yoon IY Youngstedt SD Criterion validity of the Pittsburgh Sleep Quality Index: Investigation in a non-clinical sample. Sleep Biol Rhythms (2006) 4:129–39. 10.1111/j.1479-8425.2006.00207.x PMC3399671 22822303

[69] Ait-Aoudia M Levy PP Bui E Insana S de Fouchier C Germain A Validation of the French version of the Pittsburgh Sleep Quality Index Addendum for posttraumatic stress disorder. Eur J Psychotraumatol (2013) 4:19298. 10.3402/ejpt.v4i0.19298 PMC3773169 24044071

[70] Terwee CB Jansma EP Riphagen II de Vet HC Development of a methodological PubMed search filter for finding studies on measurement properties of measurement instruments. Qual Life Res (2009) 18:1115–23. 10.1007/s11136-009-9528-5 PMC2744791 19711195

